# Supplementary material for: The Association between HDL-C and Subclinical Atherosclerosis Depends on CETP Plasma Concentration: Insights from the IMPROVE Study
Source: Biomedicines. 2021 Mar 11;9(3):286. doi: 10.3390/biomedicines9030286 (PMC7999018; doi:10.3390/biomedicines9030286)
Supplement: Supplementary file 1 [file biomedicines-09-00286-s001.pdf]

biomedicines

MDPI

Supplementary Data

The association between HDL-C and subclinical atherosclerosis depends on CETP plasma concentration: insights from the IMPROVE study

Gualtiero I. Colombo[a,1,*], Vanessa Bianconi[b,1], Alice Bonomi[a], Sara Simonelli[c], Mauro Amato[a], Beatrice Frigerio[a], Alessio Ravani[a], Cecilia Vitali[c,d], Daniela Sansaro[a], Daniela Coggi[c], Massimo R. Mannarino[b], Kai P. Savonen[e], Sudhir Kurl[f], Bruna Gigante[g], Andries J. Smit[h], Philippe Giral[i], Elena Tremoli[a], Laura Calabresi[c], Fabrizio Veglia[a], Matteo Pirro[b,2], Damiano Baldassarre[a,l,2], on behalf of the IMPROVE Study Group†

[1]Contributed equally to this work as first authors. [2]Contributed equally to this work as last authors.

[a] Centro Cardiologico Monzino IRCCS, Milan, Italy.
[b] Department of Medicine, University of Perugia, Perugia, Italy.
[c] Department of Pharmacological and Biomolecular Sciences, Università degli Studi di Milano, Milan, Italy.
[d] Department of Medicine, University of Pennsylvania, Philadelphia, USA.
[e] Foundation for Research in Health Exercise and Nutrition, Kuopio Research Institute of Exercise Medicine, Kuopio, Finland.
[f] Institute of Public Health and Clinical Nutrition, University of Eastern Finland, Kuopio, Finland.
[g] Department of Medicine, Karolinska Institutet, Stockholm, Sweden.
[h] Department of Medicine, University Medical Center Groningen, Groningen, the Netherlands.
[i] Department of Endocrinology, Metabolism, and Prevention of Cardiovascular Diseases, Pitié-Salpêtrière Hospital - Sorbonne University, Paris, France.
[l] Department of Medical Biotechnology and Translational Medicine, Università degli Studi di Milano, Milan, Italy.

**Corresponding author:*
Gualtiero I. Colombo, MD. PhD
Unit of Immunology and Functional Genomics
Centro Cardiologico Monzino IRCCS
Via Carlo Parea, 4; 20138 Milan – Italy
Telephone: +39 02-58002464 – Fax: +39 02-58002750
E-mail address: gualtiero.colombo@cardiologicomonzino.it

†The IMPROVE study group

- Centro Cardiologico Monzino, IRCCS, Milan Italy: E. Tremoli, D. Baldassarre, M. Amato, B. Frigerio, A. Ravani, D. Sansaro, D. Coggi, F. Veglia, C. Tedesco, N. Capra, A. Bonomi.
- Department of Medical Biotechnology and Translational Medicine, Università di Milano, Milan, Italy: D. Baldassarre.
- Dipartimento di Scienze Farmacologiche e Biomolecolari, Università di Milano, Milan, Italy: Laura Calabresi, C.R. Sirtori.
- Cardiovascular Medicine Unit, Department of Medicine Solna, Karolinska Institutet. Per Eriksson, Angela Silveira, Bruna Gigante.
- Division of Cardiovascular and Nutritional Epidemiology, Institute of Environmental Medicine, Karolinska Institutet. Karin Leander, Federica Laguzzi.
- Cardiovascular Medicine Unit, Department of Medicine Solna, Karolinska Institutet. Anders Hamsten.
- Division of Cardiovascular and Nutritional Epidemiology, Institute of Environmental Medicine, Karolinska Institutet. Ulf de Faire.
- Cardiovascular Genetics, Institute Cardiovascular Science, University College of London, Rayne Building, University Street, London, United Kingdom. Steve E. Humphries, J. Cooper, J. Acharya.
- Foundation for Research in Health Exercise and Nutrition, Kuopio Research Institute of Exercise Medicine, Kuopio, Finland: K. Savonen, K. Huttunen, E. Rauramaa, I.M. Penttila, J. Törrönen.
- Department of Medicine, University Medical Center Groningen, Groningen & Isala Clinics Zwolle, Department of Medicine; the Netherlands: Andries J. Smit, A.I. van Gessel, A.M van Roon, A. Nicolai, D.J. Mulder, G.H. Smeets.
- Sorbonne Université, INSERM UMR1166, cardiovascular prevention unit, AP-HP, Groupe Hôpitalier Pitié-Salpetriere, Paris, France: Philippe Giral, Anatole Kontush, Alain Carrié, Antonio Gallo.
- Institute of Public Health and Clinical Nutrition, University of Eastern Finland, Kuopio Campus: Sudhir Kurl, J. Karppi, T. Nurmi, K. Nyyssönen, T.P. Tuomainen, J.Tuomainen, J. Kauhanen.
- Internal Medicine, Angiology and Arteriosclerosis Diseases, Department of Medicine, University of Perugia, Perugia, Italy: M. Pirro, M.R. Mannarino, G. Vaudo, V. Bianconi, D. Siepi, G. Lupattelli.

Supplementary Tables and Figures

Supplementary Table S1

Association between CETP plasma concentration and cIMT variables in the cohort of drug-free subjects (n = 552).

	Unadjusted analysis		Adjusted analysis	
	β ± SE	*p*-value	β ± SE	*p*-value
$cIMT_{max}$	0.011 ± 0.021	0.61	0.015 ± 0.02	0.45
$cIMT_{mean\text{-}max}$	0.026 ± 0.020	0.18	0.032 ± 0.018	0.08
PF $CC\text{-}IMT_{mean}$	-0.004 ± 0.011	0.73	-0.002 ± 0.011	0.87

Beta values (β) are expressed in standard deviation units. Results of analyses not adjusted and adjusted for age, sex, HDL-C, log-triglycerides, total cholesterol, and latitude are presented.
SE, standard error; HDL-C, high-density lipoprotein cholesterol.

Supplementary Table S2

Stratifying drug-free patients according to the levels of HDL-C (1st, 2nd, and 3rd quartiles *vs.* top quartile) and CETP (above or below the median).

	CETP < 1.38 µg/mL (median)	CETP ≥ 1.38 µg/mL (median)
HDL-C < 58 mg/dL (1st, 2nd, 3rd quartiles), *n* (%)	214 (78.1)	199 (71.6)
HDL-C ≥ 58 mg/dL (top quartile), *n* (%)	60 (21.9)	79 (28.4)

Data are expressed as counts (n) and percentage (%).
CETP, cholesteryl ester transfer protein; HDL-C, high-density lipoprotein cholesterol.

Supplementary Table S3

CETP and HDL-C plasma concentrations stratified according to the genotype at *CETP* SNPs associated with $cIMT_{max}$ in individuals free of any pharmacological treatment.

SNP	*n*	CETP (µg/mL)	*p* for trend	HDL-C (mg/dL)	*p* for trend
**rs12444708**					
CC	403	1.39 ± 0.31	0.09	49.1 ± 14.3	0.04
TC	125	1.44 ± 0.34		51.0 ± 16.9	
TT	9	1.44 ± 0.35		59.9 ± 19.8	
**rs9938160**					
TT	294	1.39 ± 0.32	0.2	48.7 ± 14.4	0.06
CT	207	1.42 ± 0.33		50.8 ± 16.1	
CC	36	1.44 ± 0.30		52.5 ± 14.2	
**rs72786786**					
GG	284	1.40 ± 0.32	0.78	47.1 ± 13.8	**<0.001**
AG	206	1.41 ± 0.33		52.0 ± 16.1	
AA	47	1.40 ± 0.31		56.1 ± 14.6	
**rs173539**					
CC	281	1.39 ± 0.31	0.65	47.0 ± 13.4	**<0.001**
TC	209	1.41 ± 0.35		52.1 ± 16.6	
TT	47	1.40 ± 0.30		55.7 ± 14.1	
**rs34760410**					
CC	439	1.40 ± 0.33	0.54	49.4 ± 14.8	0.2
TC	96	1.42 ± 0.29		50.8 ± 16.6	
TT	2	1.41 ± 0.07		68.7 ± 10.0	
**rs12920974**					
GG	273	1.41 ± 0.33	0.92	50.3 ± 14.6	0.24
TG	225	1.39 ± 0.31		49.5 ± 15.6	
TT	39	1.45 ± 0.32		47.2 ± 15.5	
**rs3764261**					
CC	290	1.39 ± 0.31	0.75	46.7 ± 13.5	**<0.001**
AC	200	1.41 ± 0.34		52.7 ± 16.6	
AA	47	1.41 ± 0.30		56.2 ± 13.9	
**rs12708968**					
TT	422	1.40 ± 0.33	0.8	49.6 ± 14.8	0.49
CT	112	1.41 ± 0.30		50.4 ± 16.2	
CC	3	1.36 ± 0.11		55.0 ± 23.9	
**rs12708974**					
CC	415	1.40 ± 0.33	0.78	49.5 ± 14.8	0.37
TC	117	1.40 ± 0.31		50.7 ± 16.1	
TT	5	1.53 ± 0.15		52.3 ± 15.3	

Values of CETP concentration and HDL-C are expressed as mean ± SD; *p* for trend values in bold were statistically significant after Bonferroni correction for multiple comparisons. CETP, cholesteryl ester transfer protein; HDL-C, high-density lipoprotein cholesterol; SNP, single-nucleotide polymorphism.

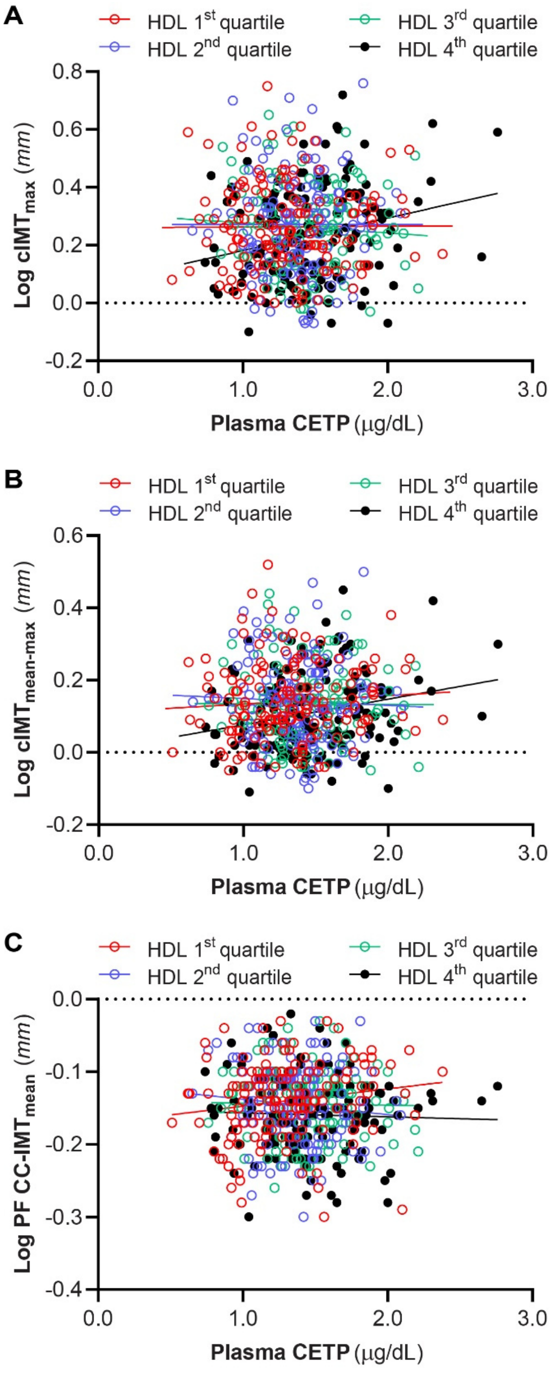

	F	*p*-value
HDL quartiles	5.419	0.020
CETP plasma concentration	2.330	0.127
HDL quartiles × plasma CETP	5.717	**0.017**

	F	*p*-value
HDL quartiles	5.534	0.019
CETP plasma concentration	1.109	0.293
HDL quartiles × plasma CETP	4.113	**0.043**

	F	*p*-value
HDL quartiles	0.089	0.766
CETP plasma concentration	0.459	0.498
HDL quartiles × plasma CETP	0.712	0.399

Supplementary Figure S1. Correlations between CETP plasma concentration and cIMT variables in the different quartiles of HDL-C.

(A) Correlations with $cIMT_{max}$, (B) with $cIMT_{mean\text{-}max}$, and (C) with PF $CC\text{-}IMT_{mean}$. The interaction CETP concentration × HDL-C is adjusted for age, sex, latitude, total cholesterol, and log-triglycerides.

HDL, high-density lipoprotein; CETP, cholesteryl ester transfer protein; $cIMT_{max}$, highest carotid intima-media thickness value among common carotids, bifurcations, and internal carotid arteries; $cIMT_{mean\text{-}max}$, average of 8 maximal cIMT measures; PF $CC\text{-}IMT_{mean}$, average of plaque-free areas of common carotids.
